# Synthesis and Characterization of *trans*-Dichlorotetrakis(imidazole)cobalt(III) Chloride: A New Cobalt(III) Coordination Complex with Potential Prodrug Properties

**DOI:** 10.1155/2018/4560757

**Published:** 2018-09-03

**Authors:** Kaila F. Hart, Natalie S. Joe, Rebecca M. Miller, Hannah P. Nash, David J. Blake, Aimee M. Morris

**Affiliations:** ^1^Department of Chemistry and Biochemistry, Fort Lewis College, 1000 Rim Dr., Durango, CO 81301, USA; ^2^Department of Biology, Fort Lewis College, 1000 Rim Dr., Durango, CO 81301, USA

## Abstract

Numerous therapies for the treatment of cancer have been explored with increasing evidence that the use of metal-containing compounds could prove advantageous as anticancer therapeutics. Previous works on Ru(III) complexes suggest that structurally similar Co(III) complexes may provide good alternative, low-cost, effective prodrugs. Herein, a new complex, *trans*-[Co(imidazole)_4_Cl_2_]Cl (**2**), has been synthesized in high yields utilizing ligand exchange under refluxing conditions. The structure of **2** has been characterized by elemental analysis, ^1^H and ^13^C·NMR, ESI-MS, CV, and UV-Vis. The ability of **2** to become reduced in the presence of ascorbic acid was probed demonstrating the likely reduction of the Co(III) metal center to Co(II). In addition, preliminary cell line testing on **2** shows a lack of cytotoxicity.

## 1. Introduction

The use of metals or metal-containing complexes for the treatment of cancer dates back to the sixteenth century but is still considered a relatively unexplored area of cancer research [1]. Increasing evidence suggests that the use of metal-containing compounds could prove to be advantageous as anticancer therapeutics [2, 3]. The use of metal complexes in anticancer therapies offers several advantages including the use of multiple oxidation states of metals, a larger range of geometries to be explored and utilized, and a so-called “tunability” of the thermodynamics and kinetics of ligand substitution [4–8].

**Figure 1:**
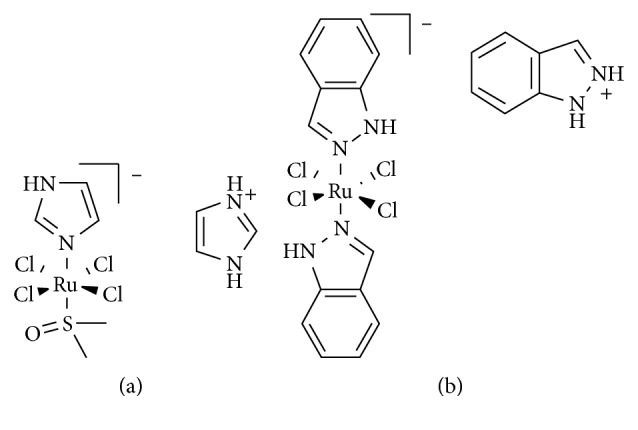
Structures of (a) NAMI-A and (b) KP1019.

Cisplatin targets DNA and has been successful for the treatment of many different cancers but has the disadvantage of being toxic to both healthy and affected tissues [9]. In a movement to develop less-toxic anticancer drugs, recent attention has been focused on the use of prodrugs. A prodrug remains inactive until it reaches diseased cells at which time it becomes activated and cytotoxic. To date, the most successful and studied metal-containing prodrugs are so-called functional models with Ru(III) centers, NAMI-A [10, 11] and KP1019 [12, 13] (Figure 1). Functional models refer to the “naked” metal center performing the desired therapeutic function as both have mounting evidence to strongly suggest loss of their original ligands and can complex to other Lewis bases *in vivo* [14–17]. While the mechanism of action for NAMI-A and KP1019 is still unknown [11, 18, 19], recent studies on NAMI-A and derivatives of KP1019 suggest that the ruthenium center can coordinate to nitrogen, oxygen, or sulfur amino acid donors on protein targets [15, 20–22].

It has been suggested that Co(III) complexes, with their octahedral geometries and low-spin *d* ^6^ configurations, may also represent an effective class of prodrugs for the treatment of cancer [2, 3, 20–23]. In addition, cobalt is ca. 30,000 times more abundant in the earth's crust and 1.2% of the current cost of ruthenium [24]. The octahedral, low-spin *d* ^6^ electron configurations give rise to diamagnetic complexes that can easily be assigned and followed using nuclear magnetic resonance (NMR) spectroscopy. Substantial research has been published involving the synthesis and characterization of Co(III) complexes with one or more bidentate or polydentate ligands [8, 9, 25–30], having the potential disadvantage of the kinetic chelate effect if open sites on the metal center are needed to induce apoptosis. However, the synthesis of functional models of Co(III) complexes similar in structure to NAMI-A or KP1019 have not been reported and provide a relatively unexplored realm for anticancer prodrug therapies.

Herein, the synthesis of a new potential Co(III) functional prodrug, *trans*-dichlorotetrakis(imidazole)cobalt(III) chloride (**2**) is presented utilizing a ligand substitution reaction from a known Co(III) starting material, *trans*-dichlorotetrakis(pyridine)cobalt(III) chloride (**1**) [31, 32]. Characterization of **2** by ESI-MS, IR, ^1^H and ^13^C·NMR, UV-Vis, elemental analysis, and cyclic voltammetry all support the proposed structure. Furthermore, the characterization and preliminary biological studies of **2** suggest its potential as a prodrug.

## 2. Experimental

### 2.1. Materials and Methods

Chlorine (≥99.5%) and anhydrous pyridine (99.5 + %) were purchased from Sigma-Aldrich and Alfa Aesar, respectively, and used as received. Cobalt(II) chloride hexahydrate (98–102%; Alfa Aesar), imidazole (97 + %; Alfa Aesar), methyl isobutyl ketone (EMSURE), dimethyl sulfoxide (Fisher), and diethyl ether (Fisher) were of ACS grade and used as received without further purification.

Mass spectra were collected using a Finnigan LCQ Deca XP Plus ion trap with ions generated using an electrospray ionization (ESI) source with a spray voltage of 4.5 kV, heated capillary temperatures of 200–250°C, and a flow of 10* μ*L/min. The electrospray solution contained ∼10^−5^ M of **2** in CH_3_CN (anhydrous, 99.8%; Sigma-Aldrich). Elemental Analysis was performed by Galbraith Laboratories, Inc., Knoxville, TN. The ^1^H and ^13^C·NMR spectra were run on a JEOL ECX-400 spectrometer, operating at 400 MHz and 100 MHz, respectively. NMR spectra were run in DMSO-*d*_6_ (Acros; 99.6%) or CDCl_3_ (Acros; 99.8%) dried over activated molecular sieves, or D_2_O (Cambridge Isotope Laboratories; 99.9%). NMR spectra were referenced to the residual solvent peak. FTIR in the solid state was taken on the Thermo Scientific Nicolet iS10 with Smart iTR from 600–4000 cm^−1^.

### 2.2. Electrochemistry

Cyclic voltammetry (CV) was carried out with a BASi epsilon digital potentiostat at a scan rate of 100 mV/s using a glassy carbon working electrode, a platinum auxiliary electrode, and an Ag/AgCl reference electrode. The solution for analysis contained 1 mM of **2** in DMSO with 0.1 M tetraethylammonium perchlorate. The solution was degassed with nitrogen prior to running CV at room temperature, using a ferrocene internal standard (Fc/Fc^+^ = +0.68 V in DMSO).

### 2.3. Synthesis of Cobalt(III) Starting Material, *trans*-Dichlorotetrakis(pyridine)cobalt(III) Chloride, **1**

The synthesis of this starting material was achieved using a modified version of literature procedures [31, 32] (see Figure S1 of the Supplementary Materials for a detailed experimental setup). Specifically, 10.0 g (0.042 mol) of cobalt(II) chloride hexahydrate was solubilized in 40.0 mL of distilled water in a round-bottom flask with stirring and 30°C heating resulting in a magenta-colored solution. Next, 13.0 mL (0.161 mol) of pyridine was added dropwise (∼1 drop/second) resulting in a final deep red/purple solution. The round-bottom flask was sealed with a septum and oxidized with vigorous bubbling of chlorine gas at 30°C for thirty minutes. The final dark brown solution with a visible solid green product present was then purged with N_2_ for 20 minutes before being placed in the refrigerator for 1 week. The desired flaky green product was collected over a medium filter frit and washed 3 times with ∼10 mL portions of ice-cold water, followed by ice-cold ethanol and ice-cold ether. The product was dried overnight on a high vacuum line. Yield: 5.0 g (25%). ^1^H·NMR (400 MHz, CDCl_3_, *δ*): 7.42 (t, *J* = 6.6 Hz, 2H), 8.08 (t, *J* = 7.2 Hz, 1H), and 8.42 (*d*, *J* = 4.8 Hz, 2H).

### 2.4. Synthesis of *trans*-Dichlorotetrakis(imidazole)cobalt(III) Chloride, **2**

In a 50 mL round-bottom flask, 0.10 g (0.21 mmol) of **1** was partially solubilized in 10 mL of methyl isobutyl ketone with stirring and slight heating (40°C). In a beaker, 0.071 g (1.0 mmol) of imidazole was fully solubilized in 20 mL of methyl isobutyl ketone with stirring. The solubilized imidazole was added to the 50 mL round-bottom flask. The initial solution is light green with green particles visible in the solution. A water condenser was attached, and the solution was refluxed at 115°C for 2 hours. During the reflux, the starting material becomes fully solubilized. After the reflux period, a new green solid in blue solution is observed in the round-bottom flask. The solid was collected on a medium filter frit and washed three times with ∼5 mL portions of ice-cold methyl isobutyl ketone followed by 3 × 5 mL portions of ice-cold diethyl ether. Yield: 0.070 g (77%). Note that this procedure has been reproducibly scaled up by 10 times maintaining reported yields with an increased reflux time of 24 hours.

### 2.5. Analytical Data for Complex **2**

[Co(C_3_H_4_N_2_)_4_Cl_2_]Cl (437.60 g/mol): calculated [found]: C, 32.94 [33.66]; H, 3.69 [4.06]; and N, 25.61 [24.70]. MS (ESI): *m*/*z* 401 [M^+^]. FTIR (cm^−1^): *ν*(N–H) 3135; *ν*(C–H) 3086, 2975, and 2871; *ν*(C=N/C=C) 1551, 1500, 1450, and 1334. ^1^H·NMR (DMSO-*d* ^6^) *δ*: 6.86 (*s*, 1H), 7.18 (*s*, 1H), 7.71 (*s*, 1H), and 13.1 (s, NH); ^13^C·NMR (DMSO-*d* ^6^) *δ*: 116.76, 130.47, and 140.79.

### 2.6. UV-Vis Studies of Complex **2**

Samples for UV-Vis were prepared in distilled water with 2 mM of complex **2**. Studies using nitrogen-degassed and sealed cuvette samples as well as samples in air showed no experimental difference. The ability for the reduction of the Co(III) metal center of **2** was studied by adding 10 equivalents of ascorbic acid.

### 2.7. Cell Culture, Reagents, and Cytotoxicity Studies

A549 cells (human epithelial lung carcinoma) were obtained from American Type Culture Collection (Manassas, VA). The cells were cultured at 37°C in a 5% CO_2_ incubator in complete media containing modified Eagle's media with 10% fetal bovine serum and antibiotics (100 U/mL penicillin and 10 *μ*g/mL streptomycin; Life Technologies). Complex **2** was dissolved in sterilized DMSO and diluted in cell culture media immediately before use.

Cell viability was assessed 24 h after exposure to varying concentrations of either **2** (10 nM–1 mM) or the corresponding concentration of the DMSO control through the CellTiter-Blue Cell Viability Assay (Promega). Fluorescence (560_EX_/590_EM_) was acquired using the Infinite® M200 Microplate reader (Tecan). Fluorescence at 590_EM_ is proportional to the number of viable cells.

## 3. Results and Discussion

### 3.1. Synthesis and Characterization of Complex **2**

**Scheme 1:**
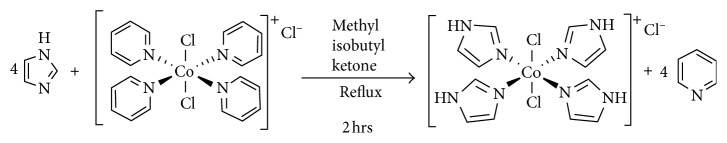
Stoichiometric equation for the formation of *trans*-[Co(imidazole)_4_Cl_2_]Cl (**2**).

Complex **2** was synthesized in 77% yield in micro- and macroscale quantities according to the stoichiometric equation outlined in Scheme 1. Under refluxing conditions, the pyridine ligands can be displaced from **1** and replaced with the desired imidazole ligands to yield the new desired product, **2**, as a green powder. Synthetically, the highest yields of pure product were obtained by using a slight excess of the imidazole ligand: five equivalents versus the stoichiometric four equivalents. The bulk solid sample composition was verified by elemental analysis and FTIR and is consistent with the assigned formula, [Co(C_3_H_4_N_2_)_4_Cl_2_]Cl. The FTIR of **2** is similar to free imidazole with the most significant change being the disappearance of hydrogen bonding vibrations that exist between 2800 and 2400 cm^−1^ in free imidazole (Figure S2 of the Supplementary Materials), as has been observed in other cobalt imidazole complexes [33, 34]. ESI-MS with collision-induced dissociation (CID) also supports the bulk composition proposed for complex **2** (Figure S3 of the Supplementary Materials).

**Figure 2:**
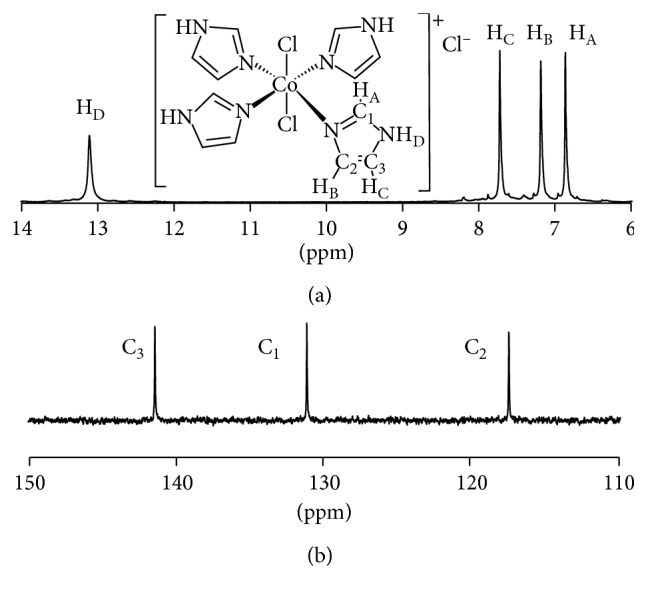
^1^H·NMR (a) and ^13^C·NMR (b) of **2** in DMSO-*d*_6_.

The octahedral, low-spin, diamagnetic Co(III) metal center allowed for straightforward characterization of **2** by ^1^H and ^13^C·NMR, providing additional support of the assigned chemical formula and structure with *trans*-chloro ligands (Figure 2; full spectra are found in Figures S4 and S5 of the Supplementary Materials). Resonance and chemical equivalency for two of the free imidazole protons (H_B_ and H_C_) and carbon atoms C_2_ and C_3_ are lost upon binding to the Co(III) center. Assigned chemical shifts were determined by 1-D NOE difference and 2-D HSQC experiments (Figures S6 and S7 of the Supplementary Materials).

**Figure 3:**
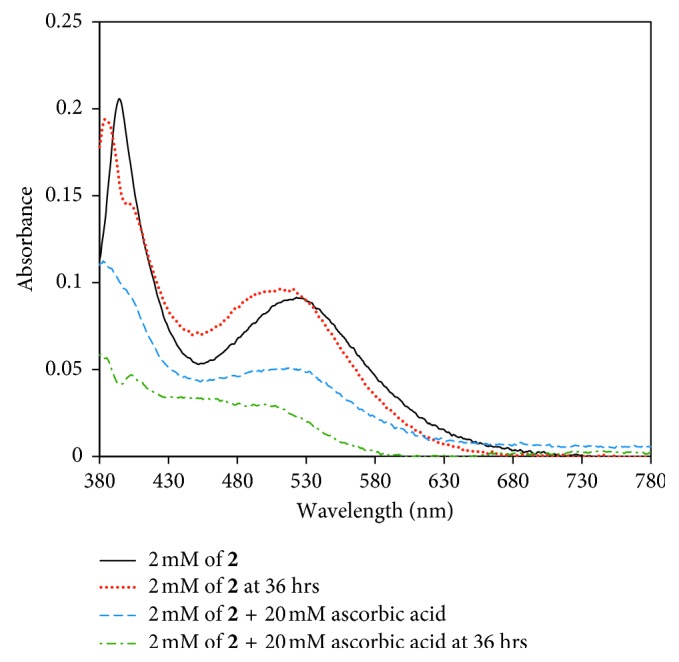
UV-Vis absorption spectra of 2 mM of **2** in H_2_O at time = 0 (black) and time = 36 hours (red). Immediately after the addition of 10 equivalents of the biological reducing agent, ascorbic acid (blue), the signature Co(III) *d*-*d* transitions decrease in intensity over time (after 36 hours is shown in green) suggesting that reduction of the cobalt center is observed.

The UV-Vis of **2** in water (black line in Figure 3) displays two maxima at 390 and 520 nm with molar absorptivity values of 100 and 47 M^−1^·cm^−1^, respectively. These are assigned as the expected tetragonally distorted *d*-*d* transitions ^1^A_1g_(D_4h_) → ^1^E_g_(D_4h_) and ^1^A_1g_(D_4h_) → ^1^A_2g_(D_4h_) of low-spin, octahedral Co(III) complexes [35]. Taken together, the ESI-MS, IR, elemental analysis, ^1^H and ^13^C·NMR, and UV-Vis of **2** all support the formation of a new Co(III) coordination complex, *trans*-dichlorotetrakis(imidazole)cobalt(III) chloride.

### 3.2. Potential Prodrug Capabilities of Complex **2**

The solubility properties of complex **2** are amenable to biological applications as **2** is soluble in polar solvents (e.g., H_2_O, CH_3_OH, and DMSO) at concentrations >10 mM. Furthermore, ligand lability is observed with complex **2** in H_2_O and D_2_O, demonstrating its potential as a functional Co(III) prodrug capable of displacing ligands.

Evidence of ligand lability in complex **2** was demonstrated by ^1^H·NMR in D_2_O. Over the course of an hour, the solution of **2** visibly changed from green to red. The time-lapsed ^1^H·NMR spectra in D_2_O (Figure S8) suggest lability of the chloro and also possibly imidazole ligands over time, demonstrating favorable characteristics for desired *in vivo* prodrug applications. The ligand lability in H_2_O was further verified by UV-Vis. In the absence of a reducing agent, **2** shows a blue shift of the two *λ*_max_ values after 36 hours likely due to ligand exchange (Figure 3 and Figure S9 of the Supplementary Materials). Furthermore, the blue shifts are indicative of a ligand exchange involving a weak-field chloro ligand being replaced by a stronger-field aqua or imidazole ligand.

**Figure 4:**
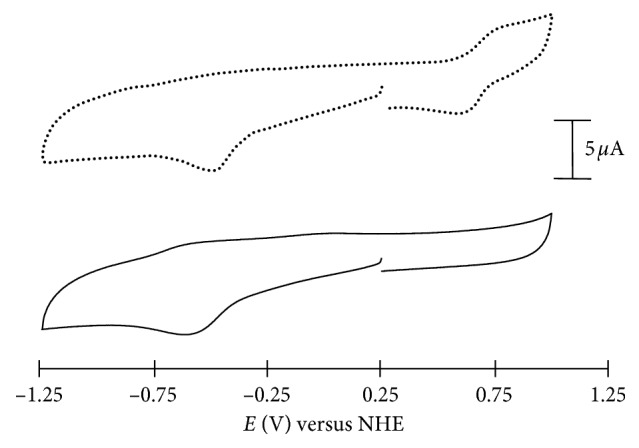
Cyclic voltammetry of 1 mM of **2** in DMSO with the internal Fc/Fc^+^ standard (top) and without (bottom).

While the “activation by reduction hypothesis” in Ru(III) prodrugs such as NAMI-A and KP1019 is still debated [11, 18, 19], exploration of biologically accessible reduction potentials and properties of potential prodrugs may provide important insights. For complex **2**, the ability of the Co(III) metal center to be reduced to Co(II) was also investigated. Previous studies indicate that a reduction potential between −0.1 V and −0.5 V versus NHE in water at pH 7 is required for successful redox cycling by common flavoproteins *in vivo* [36]. Complex **2** shows an irreversible reduction of the Co(III) metal center to Co(II) at −0.51 V versus NHE, indicating that reduction of this complex may be possible *in vivo* (Figure 4). The observed irreversibility is common in Co(III) prodrugs with multidentate ligands and is likely due to a chemical change in the structure of **2** that occurs after reduction to Co(II) [37–39].

**Figure 5:**
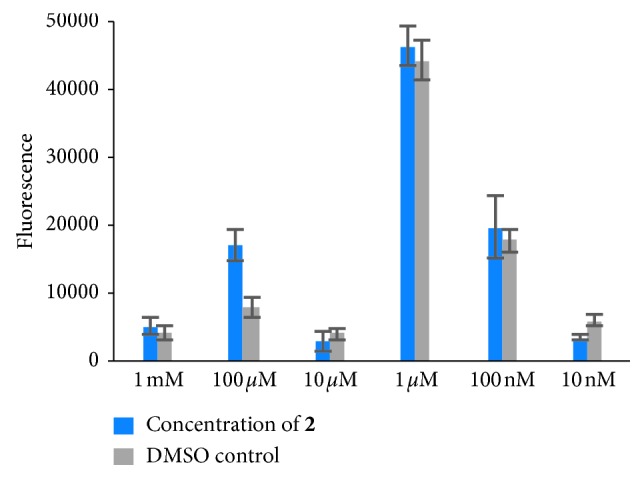
Viability of human epithelial cells is unaffected by **2**. Cells were exposed to **2** at different concentrations up to 1 mM for 24 h. Viability, which is correlated with fluorescence, was quantified using the CellTiter-Blue assay. Data presented are mean fluorescence ± SEM (*n* = 3–5 for each condition).

The desired properties displayed by **2** along with the increasing interest and potential of transition metal complexes in prodrug applications prompted us to investigate a preliminary biological study utilizing a cancerous cell line. The *in vitro* cytotoxicity of **2** at concentrations ranging from 10 nM to 1 mM was evaluated against the human A549 cell line (Figure 5). It is observed that the cells exposed to **2** (up to 1 mM) retain similar metabolic activity levels to their DMSO controls. This suggests that **2** is not cytotoxic, a result that correlates with observations of other successful metastatic prodrugs such as NAMI-A [11, 40, 41].

### 3.3. Similarities and Differences of **2** to NAMI-A and KP1019

Structural similarities of **2** to NAMI-A and KP1019 include octahedral geometries and low-spin configurations, and categorically, these are all considered kinetically inert complexes, although ligand lability is displayed in NAMI-A [11] and **2**. In addition, **2** and NAMI-A both have imidazole and chloro ligands and are very soluble in polar solvents. Admittedly, a major difference between **2** and NAMI-A and KP1019 is the overall charge on the coordination complexes; the Ru(III) complexes are overall anionic, while **2** is cationic. However, previous studies have shown that cationic (+1 and +2) octahedral Co(III) chaperone complexes are able to penetrate into hypoxic regions of tumor models [8, 35, 42] and cancerous cells [43]. Furthermore, NAMI-A has been described as “the ultimate prodrug” [11] since *in vivo* experimental evidence suggests the loss of its original ligands very quickly, suggesting the overall charge on the original coordination complex may be less important for a functional prodrug.

KP1019 displays cytotoxicity similar to traditional platinum chemotherapeutics [12, 13]. In contrast and similar to our preliminary results on **2**, prodrugs such as NAMI-A lack cytotoxicity but display favorable pharmacological properties [11, 40, 41]. It has been suggested that *in vitro* cytotoxicity may provide a measure of metal uptake but cannot predict the potential *in vivo* anticancer activity [11]. In support of this idea, evidence is mounting that the selective antimetastatic activity and observed lack of cytotoxicity of NAMI-A are the result of Ru(III)-serum albumin adduct formation [15, 16, 20]. Hence, the presented characterization and lack of cytotoxicity displayed by the new Co(III)-containing complex **2** demonstrate similarities to one of the most successful metal-based prodrugs that warrants further investigation.

## 4. Conclusions

In summary, optimal reaction conditions to synthesize a new Co(III) coordination complex, **2**, in 77% yield were discovered. All physical measurements are consistent with the reported octahedral structure of **2** as *trans*-[Co(imidazole)_4_Cl_2_]Cl. Characterization of the new complex was accomplished with ESI-MS, elemental analysis, IR, ^1^H and ^13^C·NMR, CV, and UV-Vis. Furthermore, initial evidence suggests that **2** may be suitable in prodrug applications due to its water solubility, ligand lability, ability to be reduced by biological reductants, and lack of cytotoxicity. Further biological studies on complex **2** and similar complexes are needed and represent an important new direction for future research.

## Data Availability

The data used to support the findings of this study are included within the article.

## Supplementary Materials

Supplementary Materials A single supplementary material pdf is available containing the following: a detailed experimental setup for the synthesis of *trans*-[Co(pyridine)_4_Cl_2_]Cl (**1**); FTIR of free imidazole and *trans*-[Co(imidazole)_4_Cl_2_]Cl (**2**); ESI-MS with CID of **2**; ^1^H·NMR of **2** in DMSO-*d*_6_; ^13^C·NMR of **2** in DMSO-*d*_6_; 1-D NOE difference spectra of **2**; HSQC of **2**; ^1^H·NMR of **2** in D_2_O monitored over time; and UV-Vis of **2** in H_2_O monitored over time.
