# Supplementary material for: Synthesis and Characterization of trans-Dichlorotetrakis(imidazole)cobalt(III) Chloride: A New Cobalt(III) Coordination Complex with Potential Prodrug Properties
Source: Bioinorg Chem Appl. 2018 Sep 3;2018:4560757. doi: 10.1155/2018/4560757 (PMC6140278; doi:10.1155/2018/4560757)
Supplement: Supplementary Materials — A single supplementary material pdf is available containing the following: a detailed experimental setup for the synthesis of trans-[Co(pyridine)4Cl2]Cl (1); FTIR of free imidazole and trans-[Co(imidazole)4Cl2]Cl (2); ESI-MS with CID of 2; 1H·NMR of 2 in DMSO-d 6; 13C·NMR of 2 in DMSO-d 6; 1-D NOE difference spectra of 2; HSQC of 2; 1H·NMR of 2 in D2O monitored over time; and UV-Vis of 2 in H2O monitored over time. [file 4560757.f1.pdf]

Supplementary Material for:

Synthesis and characterization of *trans*-dichlorotetrakis(imidazole)cobalt(III) chloride: a new cobalt(III) coordination complex with potential prodrug properties

Kaila F. Hart,[a] Natalie S. Joe,[a,b] Rebecca M. Miller,[a] Hannah P. Nash,[a] David J. Blake,[b] and Aimee M. Morris[a,]*

[a] Department of Chemistry and Biochemistry, Fort Lewis College, 1000 Rim Dr. Durango, CO 81301, USA
[b] Department of Biology, Fort Lewis College, 1000 Rim Dr. Durango, CO 81301, USA

Contents

Details of experimental set-up 2
IR of free imidazole and *trans*-[Co(imidazole)$_4$Cl$_2$]Cl 3
ESI-MS of *trans*-[Co(imidazole)$_4$Cl$_2$]Cl with CID 3
^{1}H NMR of *trans*-[Co(imidazole)$_4$Cl$_2$]Cl in DMSO-d$_6$ with assignments 4
^{13}C NMR of *trans*-[Co(imidazole)$_4$Cl$_2$]Cl in DMSO-d$_6$ with assignments 5
Difference NOE Spectra of *trans*-[Co(imidazole)$_4$Cl$_2$]Cl 6
HSQC with ^{13}C and ^{1}H correlations of *trans*-[Co(imidazole)$_4$Cl$_2$]Cl 7
^{1}H NMR monitored over time of *trans*-[Co(imidazole)$_4$Cl$_2$]Cl in D$_2$O 8
UV–Vis of *trans*-[Co(imidazole)$_4$Cl$_2$]Cl in H$_2$O monitored over time 9

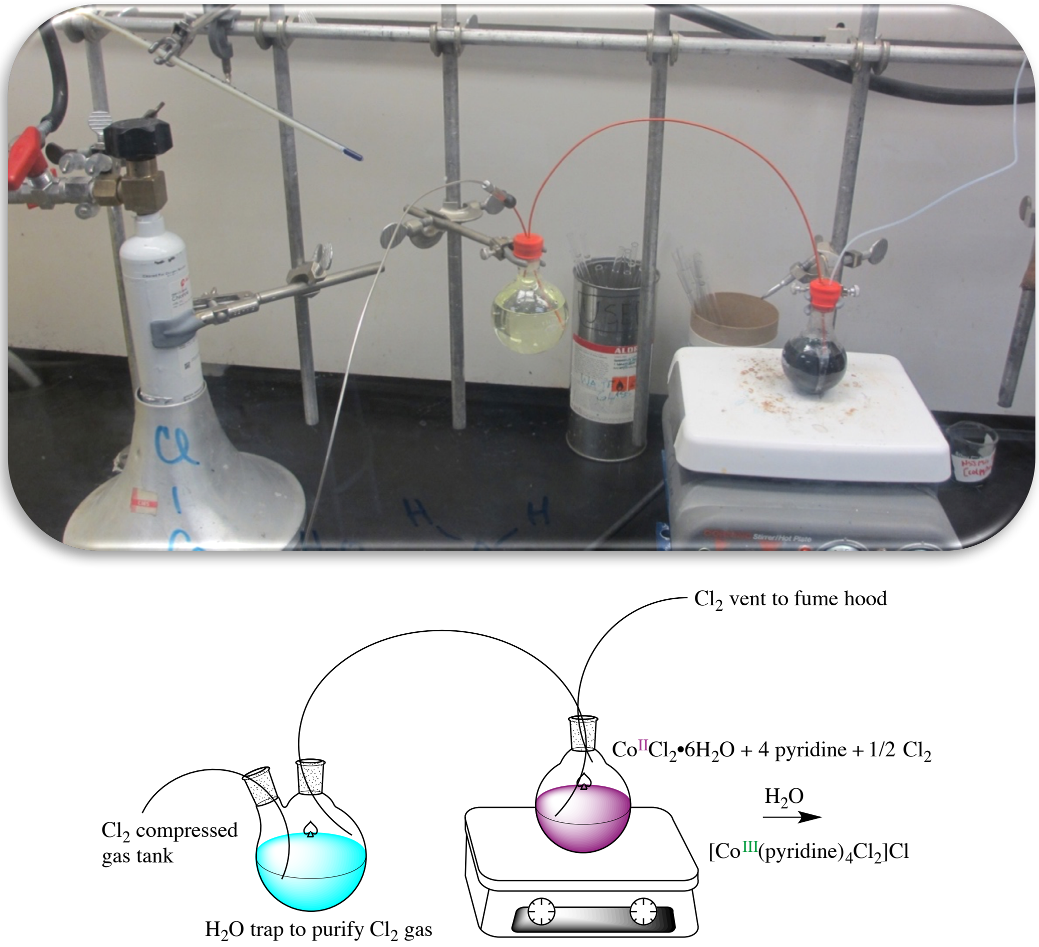

Figure S1. Experimental Set-Up for the Synthesis of *trans*-dichlorotetrakis(pyridine)cobalt(III) chloride, **1**, adapted from previous literature studies [1,2].

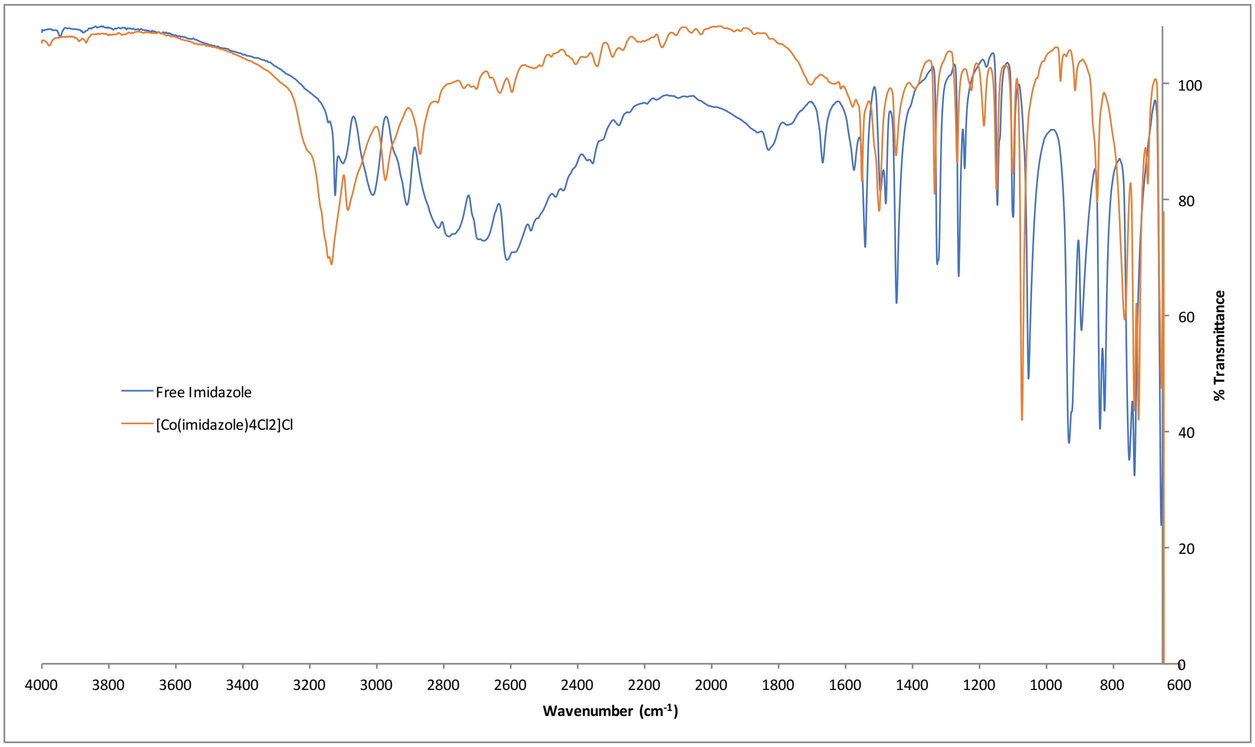

Figure S2. IR spectrum of solid free imidazole and solid *trans*-[Co(imidazole)$_4$Cl$_2$]Cl.

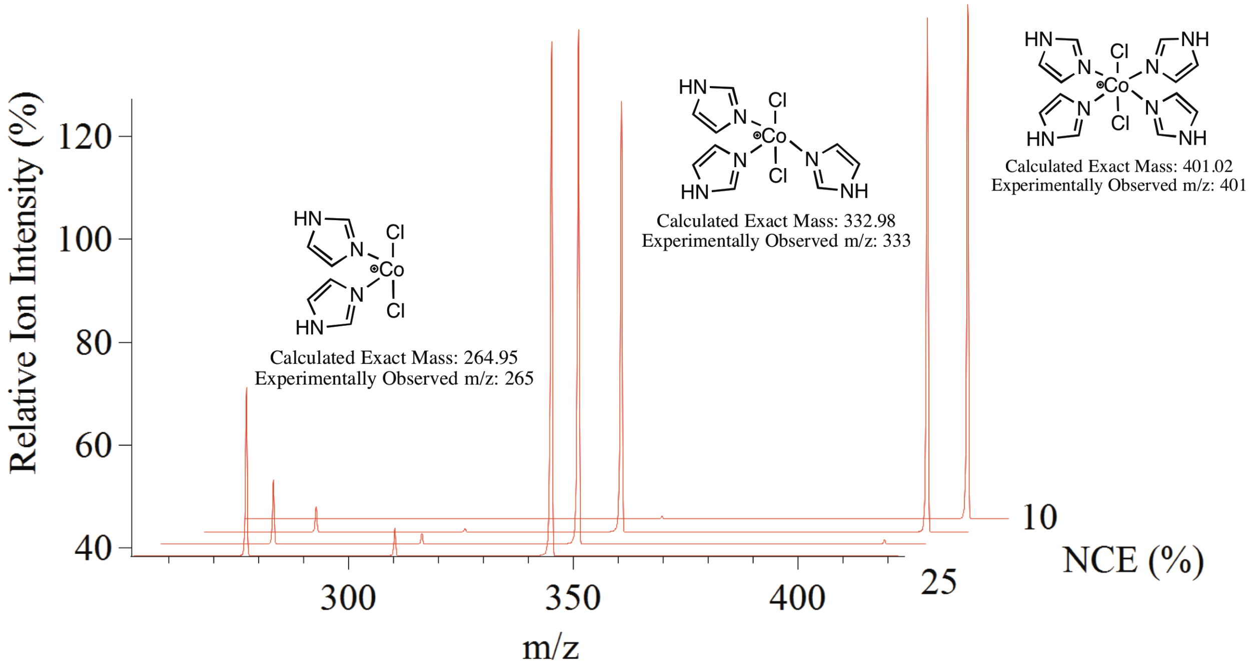

Figure S3. ESI-MS of *trans*-dichlorotetrakis(imidazole)cobalt(III) chloride by collision induced dissociation (CID) in an ion trap of the parent peak at *m/z* 401. The normalized collision energy (NCE) was 10-25%.

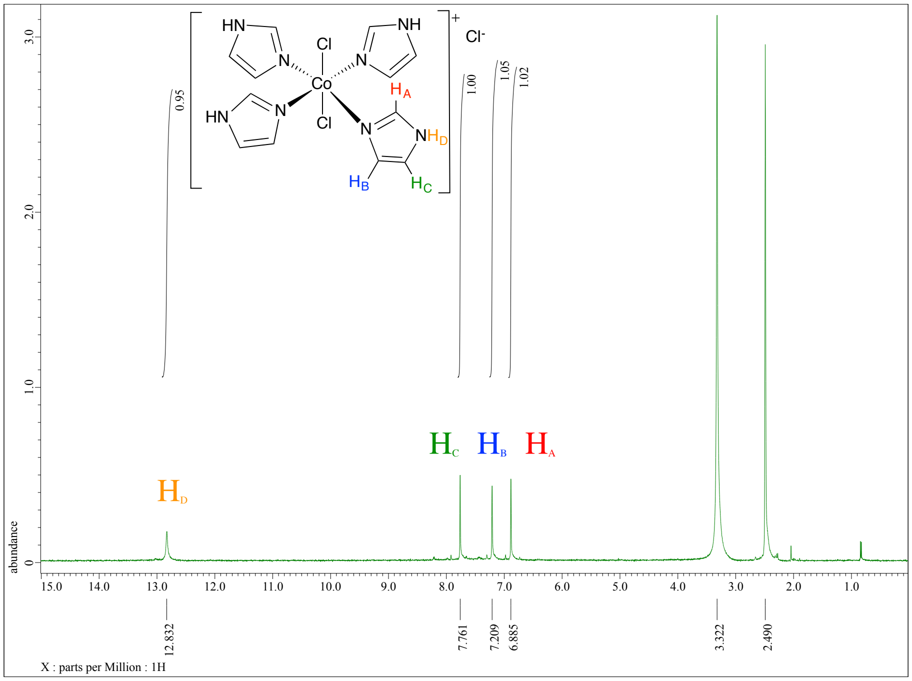

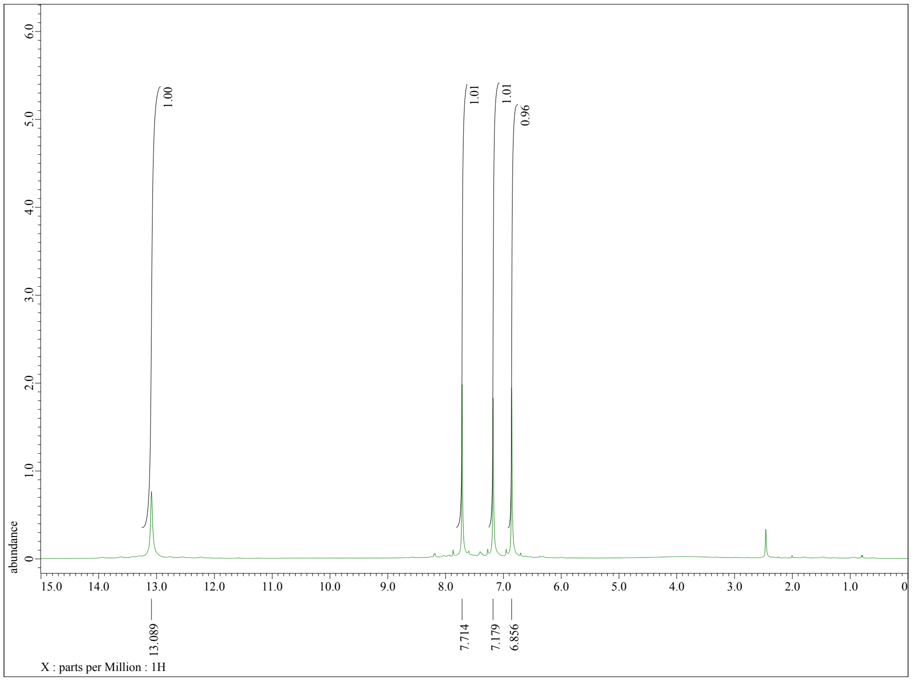

Figure S4. ^1H NMR of $[Co(imidazole)_4Cl_2]Cl$ in DMSO-d^6 initially (top) and (bottom) after 48 hours.

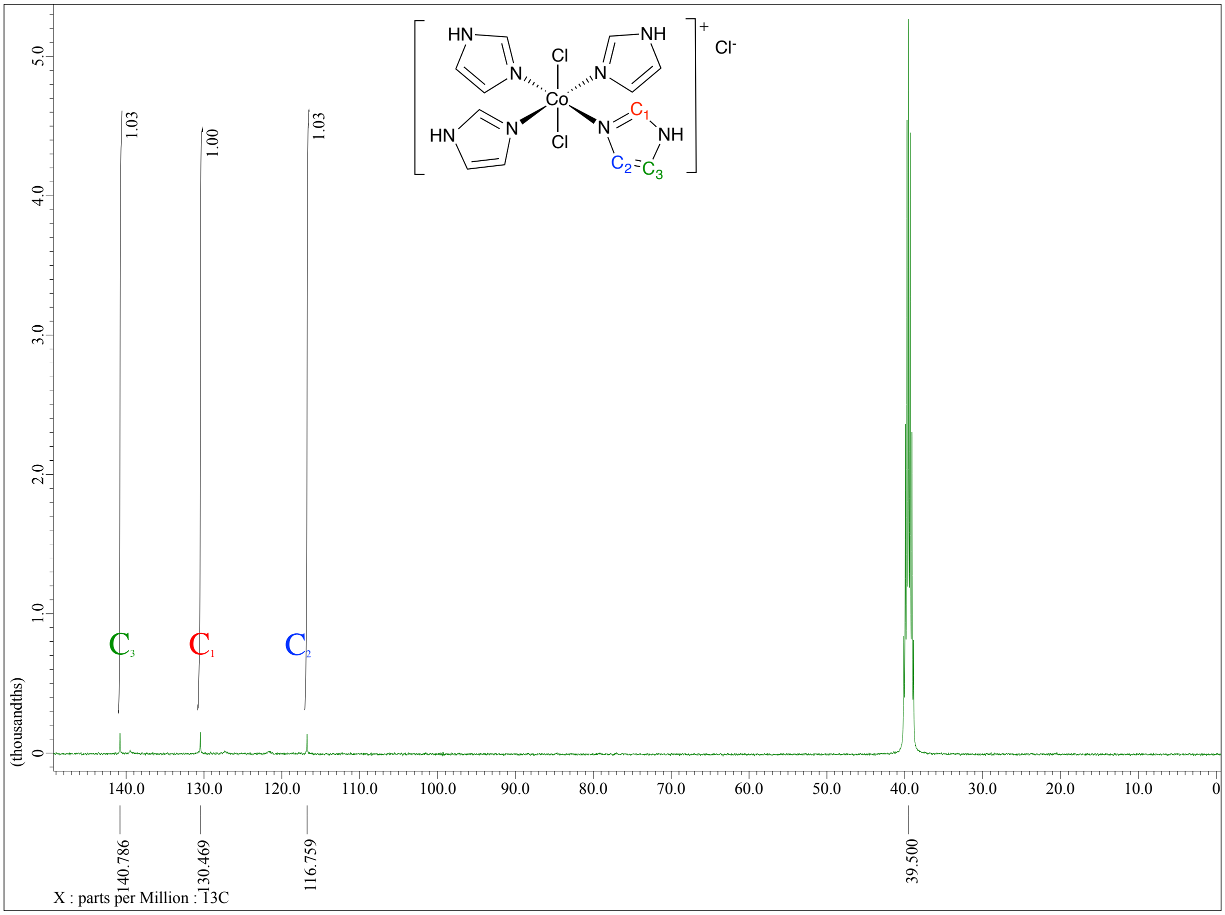

Figure S5. ^{13}C NMR of $[Co(imidazole)_4Cl_2]Cl$ in DMSO-d_6.

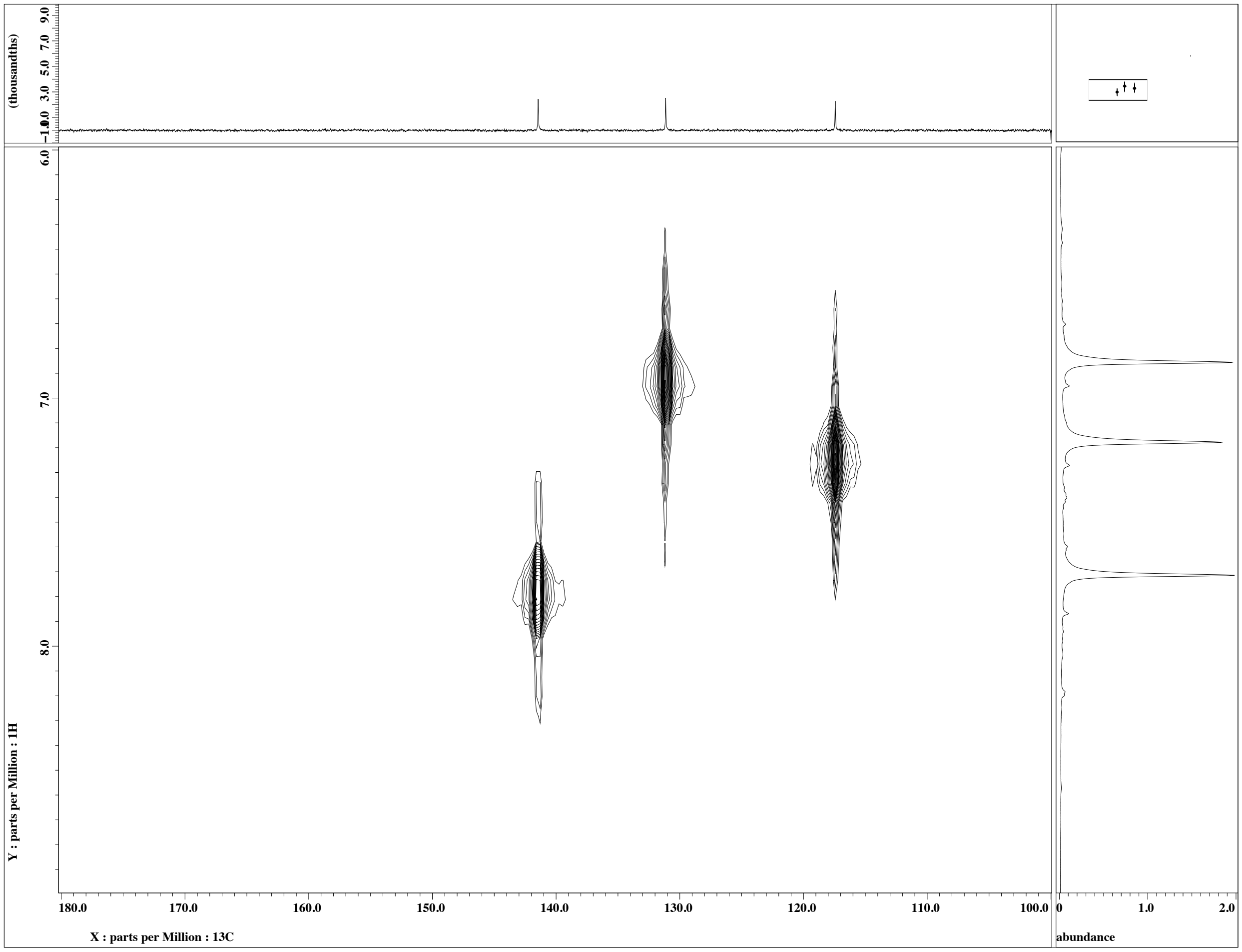

Figure S6. HSQC with ^{13}C and ^{1}H correlations of *trans*-$[Co(imidazole)_4Cl_2]Cl$ in DMSO-d_6.

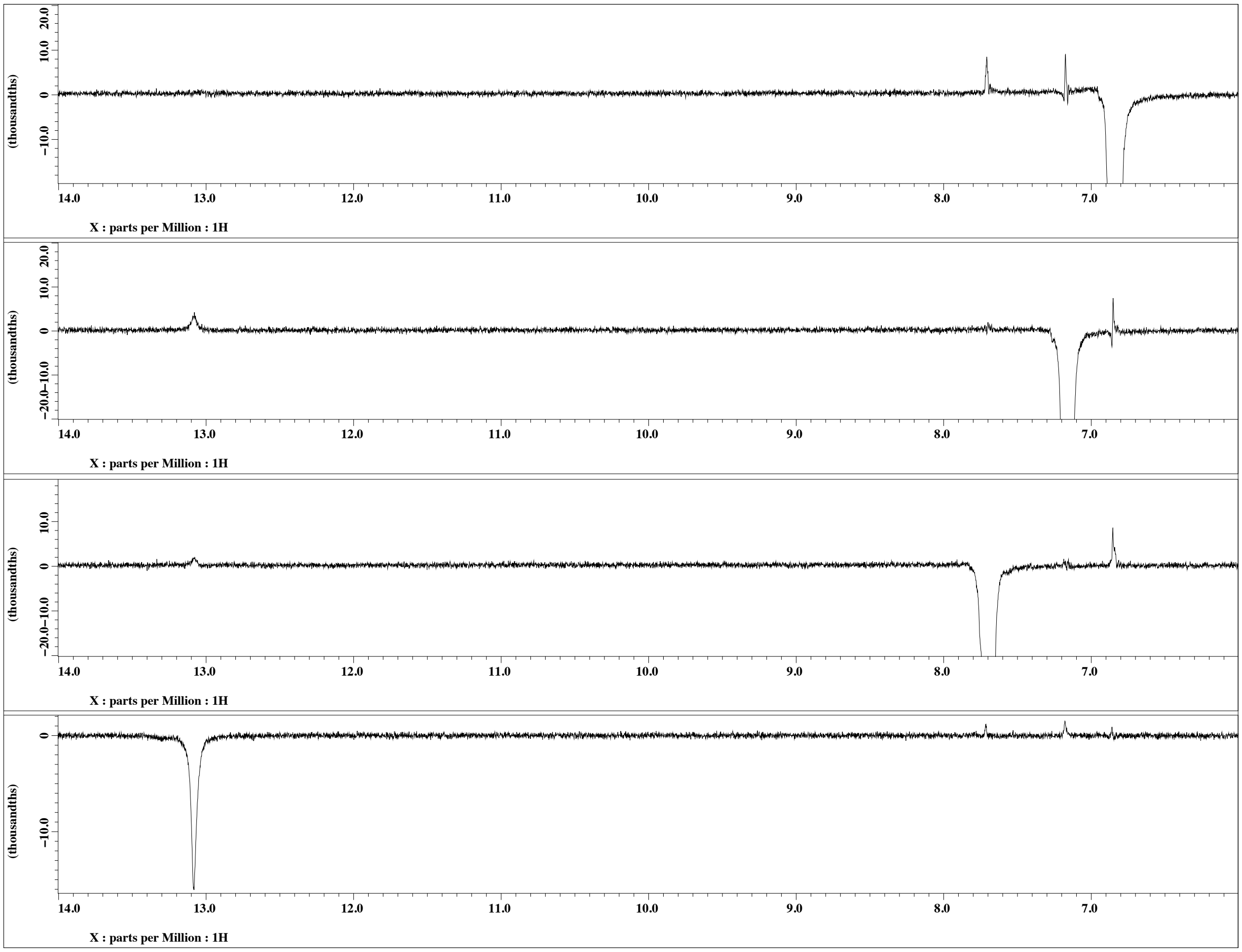

Figure S7. Difference NOE Spectra of *trans*-[Co(imidazole)$_4$Cl$_2$]Cl in DMSO-d_6.

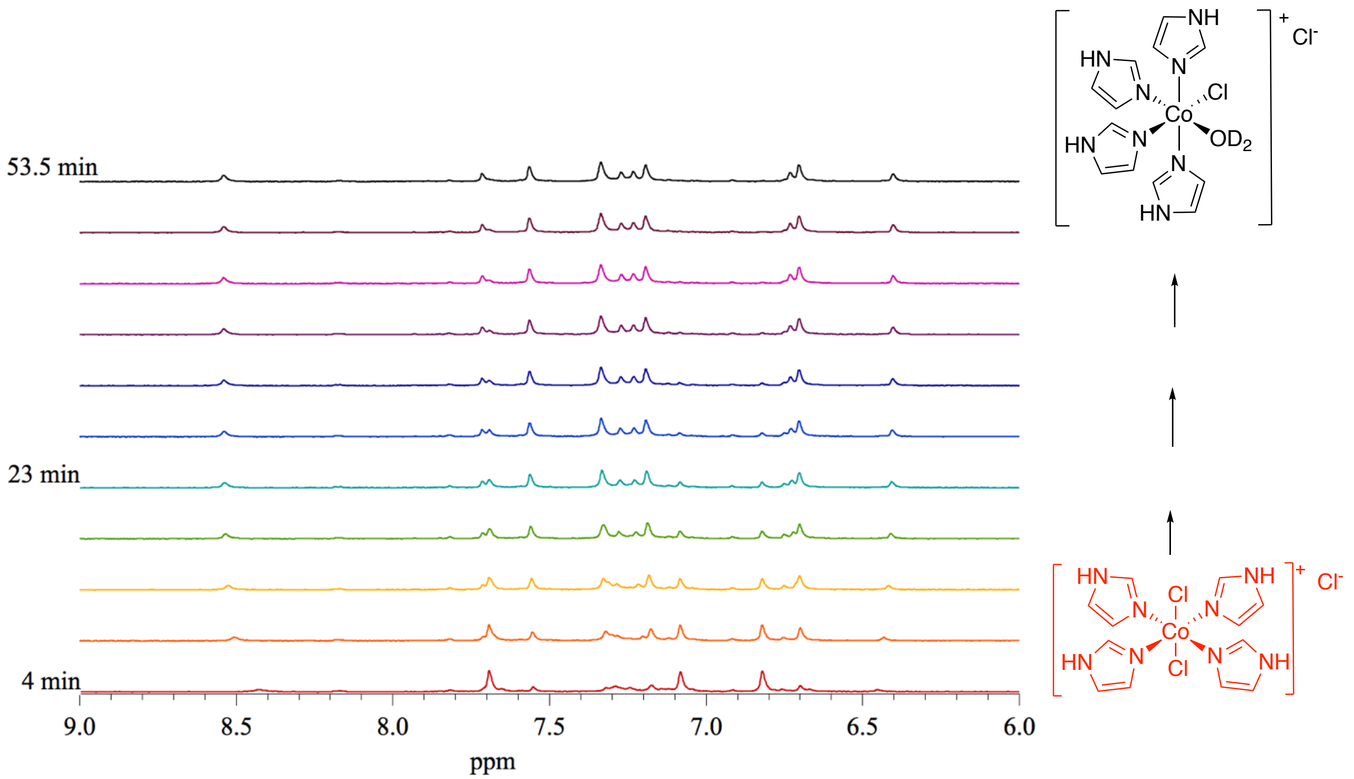

Figure S8. ^{1}H NMR of [Co(imidazole)$_4$Cl$_2$]Cl in D_2O monitored over time displaying the ligand exchange and change in structure that occurs. The original proton peaks of the bound imidazole ligands are completely gone in less than one hour. There is also no presence of free imidazole (δ D_2O: 7.62, 1H; 6.98, 2H) indicating that symmetry of the original complex is lost, but imidazole is still bound albeit likely in rearranged positions. Taken together, the ^{1}H NMR data are consistent with the starting and final structures proposed above.

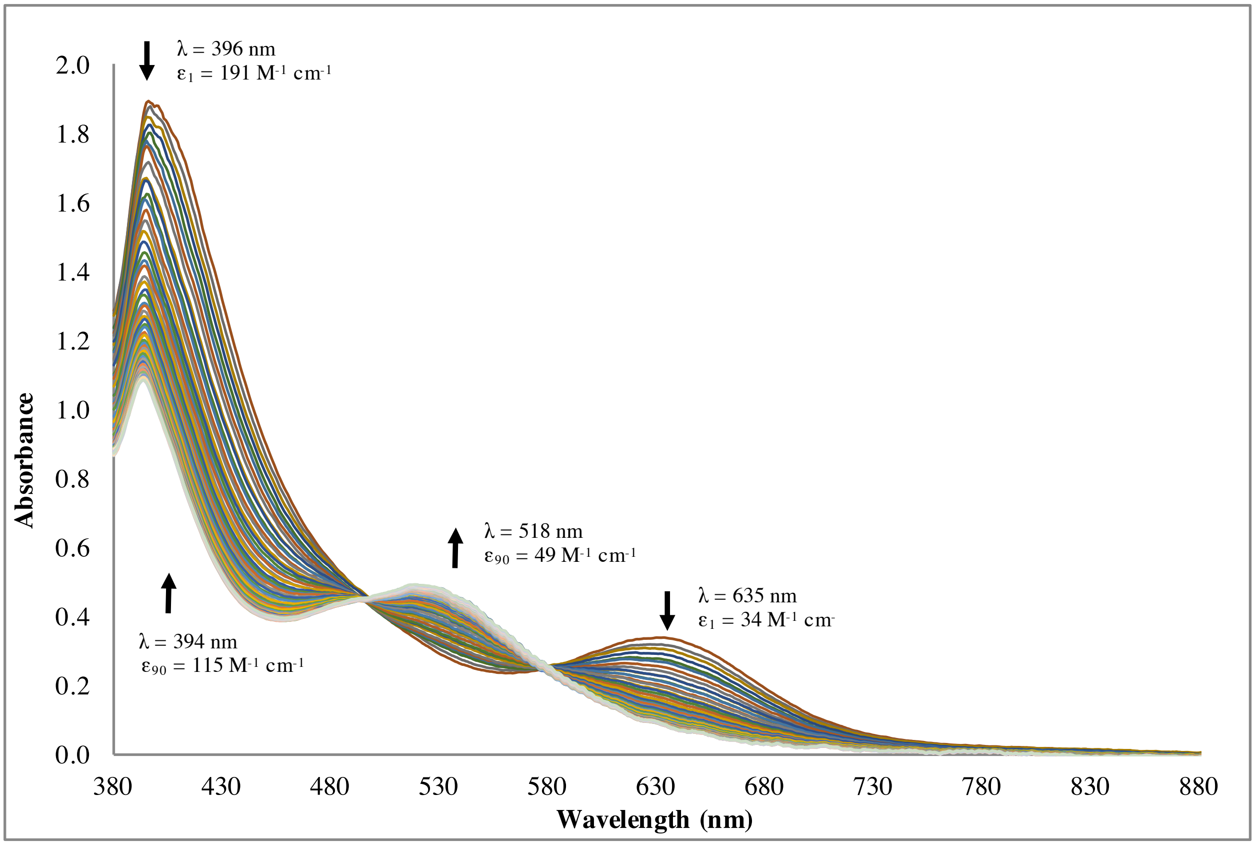

Figure S9. UV-Vis of 10 mM $[Co(imidazole)_4Cl_2]Cl$ in H_2O monitored every minute for 90 minutes.
